# Anti-ganglioside M1 autoantibodies in Egyptian children with autism: a cross-sectional comparative study

**DOI:** 10.1186/s43045-022-00202-3

**Published:** 2022-05-12

**Authors:** Reham Mohammad Raafat Hamed, Magda Ibrahim Ayoub, Mai Abdel Samie, Nancy Nabil Hamam

**Affiliations:** 1 Department of Medical Microbiology and Immunology, Faculty of Medicine, Cairo University, Kasralainy Street, Cairo, 11956 Egypt; 2 Department of Psychiatry, Faculty of Medicine, Cairo University, Kasralainy Street, Cairo, 11956 Egypt

**Keywords:** Anti-ganglioside M1 autoantibodies, Autism, Autoimmunity

## Abstract

**Background:**

Autism may be one of the pediatric autoimmune neuropsychiatric disorders, and several studies investigated the frequency of serum anti-ganglioside M1 autoantibodies in children with autism, as possible indicators of autoimmunity to the brain. The current study aimed to compare the level of anti-ganglioside M1 autoantibodies between autistic and normally developed children and to study the correlation between the level of anti-ganglioside M1 autoatibodies and the severity of autism. Forty children with autism and 40 age- and gender-matched healthy controls were enrolled. The Childhood Autism Rating Scale was used to assess the severity of autism in the patient group at the time of the study. The clinical and demographic data were recorded and plasma anti-ganglioside M1 autoantibodies level was measured in both groups.

**Results:**

The mean anti-ganglioside M1 autoantibodies level was significantly higher in autistic patients compared to the control group. The anti-ganglioside M1 autoantibodies level in patients with mild to moderate severity was insignificantly lower than its level in patients with severe autism.

**Conclusions:**

Plasma anti-ganglioside MI autoantibodies levels are higher in autistic patients than in healthy controls which may imply that some cases of autism may be autoimmune in nature.

## Background

Autism is a complex neurodevelopmental disorder generally manifesting in the first few years of life and tending to persist into adolescence and adulthood. It is characterized by deficits in communication and social interaction and restricted repetitive patterns of behavior, interests, and activities [12]. There are various screening tools for autistic disorder, including social responsiveness scale, behavioral checklist as well as the Childhood Autism Rating Scale (CARS) which is one of the most longstanding and frequently used behavior observation scales. It yields a total score, which is useful as a continuous measure of the severity of autism, as well as a categorical diagnosis of not autism, mild/moderate autism, or severe autism [21].

The exact cause of autism is unknown. It is a disorder with multifactorial etiology, there is no single cause. Some of the suspected risk factors for autism include having an intimate family member with autism, genetic mutations, fragile X syndrome and other genetic disorders, being born to older parents, low birth weight, metabolic abnormalities, exposure to heavy metals and environmental toxins, a history of viral infections, vaccines, fetal exposure to the medications valproic acid or thalidomide, and autoimmunity [24].

Genetic tests (new-generation sequencing technologies), metabolic tests (children presenting with motor delay should be evaluated with creatine kinase and thyroid-stimulating hormone testing), and immunological evaluations (as circulating maternal antibodies directed to fetal brain tissue and the potential value of maternal antibody panels) used as biomarkers of autism are currently being studied [13].

Several studies reported the production of many autoantibodies which react with specific brain structures in autistic children and affect the functions of the attacked brain tissue. Also, the potential role of maternal autoantibodies to the fetal brain in the etiology of some cases of autism has been reported [20]. Identification of brain autoantibodies as immunological biomarkers may allow earlier diagnosis of autism [11].

The exact role of anti-ganglioside M1 brain specific autoantibodies in autistic children is not yet understood [17]. Anti-ganglioside antibodies may be produced by the mechanism of molecular mimicry between gangliosides in the axon and lipooligosaccharides of the previous infectious pathogen [10]. Since autism may be one of the pediatric autoimmune neuropsychiatric disorders, so many studies aimed to investigate the frequency of serum anti-ganglioside M1 autoantibodies, as indicators of the presence of autoimmunity to the brain, in a subgroup of autistic children [17].Studies conducted by Yang et al. [17] and  and Mostafa and Al-Ayadhi [33] found that autistic children had significantly higher serum levels of anti-ganglioside M1 autoantibodies than healthy controls. Moreover, the study by Mostafa and Al-Ayadhi [17] revealed significant positive correlation with the severity of autism in autistic children. On the contrary, some studies did not show a significant difference in the rate of occurrence of anti-neuronal autoantibodies between children with autism and healthy ones [3, 23].

### Aim of the work

1. To compare the level of anti-ganglioside M1 autoantibodies in autistic and normally developed children and thus investigate the hypothesis that some cases of autism might be autoimmune in nature.
2. To study the correlation between the level of anti-ganglioside M1 autoantibodies and the severity of autism.

## Methods

This study was conducted over the period from April 2020 to April 2021. It included 80 subjects classified into 2 groups:

### Participants

#### Group 1

Forty children with autistic disorder who were following up in a specialized autism psychiatry clinic at Cairo University Hospital. This group included 7 females and 33 males with an age range from 4 to 12 years.

#### Group 2

Forty healthy control subjects without any family history of autism were collected from Abo El Reesh Preventive Medicine Pediatric Clinic for routine follow-up of growth of children. This group included 9 females and 31 males, with matched age and sex, without any neurological, immunological, or metabolic disorders. The sample size has been calculated using Hebert Arkan’s sample size equation. The sample taken was a convenient sample, where all cases and controls meeting the criteria of the study were included till the required sample number according to the sample size calculation was collected.

The patients were diagnosed according to the Diagnostic and Statistical Manual of Mental Disorders IV-text revised (DSM-IV_TR) and severity was assessed using the Childhood Autism Rating Scale (CARS).

### Procedures

Group 1 was subjected to the following:

1. Full psychiatric history-taking and examination by a specialist psychiatrist working in the Autism clinic at Cairo University Hospitals to diagnose Autistic disorder according to the Diagnostic and Statistical Manual of Mental Disorders IV-Text Revised (DSM-IV-TR) [2].
2. The Childhood Autism Rating Scale (CARS) [25] was administered by a trained clinical psychologist to assess the severity of autistic features.

Group 1 and 2 were also subjected to the following:

1. Five milliliters of blood was withdrawn from patients and control groups under complete aseptic condition and added to EDTA tubes.
2. Plasma was separated within 30 min of collection by centrifugation at 3000×*g*.
3. Samples were stored at – 20 °C.

An informed written consent was obtained from all participants.

Determination of the plasma level of anti-ganglioside M1 autoantibodies of groups 1 and 2 was performed using the ELISA Kit (Human anti-ganglioside antibody ELISA Kit, Cloud–Clone Crop, USA) for in vitro quantitative determination of anti-ganglioside antibodies in plasma. The plasma level of anti-ganglioside M1 autoantibodies of the cases’ group was compared to that of the controls’ group.

### Statistical methods

Data were coded and entered using the statistical package for the Social Sciences (SPSS) version 26 (IBM Corp., Armonk, NY, USA). Data were summarized using mean and standard deviation for quantitative variables and frequencies (number of cases) and relative frequencies (percentages) for categorical variables. Comparisons between groups were done using unpaired t-test when comparing 2 groups and analysis of variance (ANOVA) with multiple comparisons post hoc test when comparing more than 2 groups [6],or comparing categorical data, Chi-square (*χ*2) test was performed. Exact test was used instead when the expected frequency is less than 5 [7]. Correlations between quantitative variables were done using the Pearson correlation coefficient [8], *P* values less than 0.05 were considered as statistically significant***.***

## Results

This study was performed in the Faculty of Medicine Cairo University over the period from April 2020 to April 2021. Demographic data of both groups (patients and control groups) are shown in Tables 1 and 2).

**Table 1:** Comparison between the age of the patient group and control group

		***N***	Mean	SD	***P*** value
**Age**	**Autism**	40	7.43	2.96	0.693
	**Control**	40	7.68	2.67	

*NB P*value less than 0.05 is considered statistically significant

**Table 2:** Comparison between the sex of the patient group and control group

		Autism		Control		***P*** value
		Count	%	Count	%	
**Sex**	**Male**	33	82.5%	31	77.5%	0.576
	**Female**	7	17.5%	9	22.5%	

*NB P* value less than 0.05 is considered statistically significant

The group with autistic disorder consisted of 33 (82.5%) males and 7 (17.5%) females who were recruited randomly from the autism clinic at Cairo University Hospitals. The ratio of the number of males to females was ≈ 5:1.

As regards the clinical presentation, 11(27.5%) of patients presented with a defect in verbal communication skills mainly, 11 patients (27.5%) presented with a defect in non-verbal communication skills predominantly, while the remaining 18(45.0%) patients presented with both**.** Regarding autism severity, 21 (52.5%) patients showed mild to moderate severity while 19 (47.5%) patients presented with severe autism as shown in Table 3.

**Table 3:** Severity of autism according to Childhood Autism Rating Scale scores in the patients’ group

Severity of Autism	count	%
**Mild-Moderate**	21	52.5%
**Severe**	19	47.5%

*NB* *P-*value less than 0.05 is considered statistically significant

Our study aimed to estimate the level of anti-ganglioside M1 antibodies in both the patients and control groups. In this study, the level of GM1 in plasma of autistic children was higher than its level in the healthy control group (3.75 ± 0.62 ng/ml, 1.96 ± 0.3 ng/ml respectively) and this difference was statistically significant *(P* value *≤* 0.001*)*.

The anti-ganglioside M1 autoantibodies concentration in severe cases (3.77 ± 0.68 ng/ml) was slightly higher than that in mild and moderate cases (3.73 ± 0.57 ng/ml); however, this difference was not statistically significant (*P* value = 0.840).

## Discussion

Autoimmune diseases are characterized by aberrant chronic activation of the immune system which causes tissue inflammation and damage in genetically predisposed individuals [34]. Some studies suggest associations between immune system abnormalities or cytokine aberrations with autism, suggesting that immunological dysregulation is an important comorbidity of autism and may play a role in its pathogenesis, including up regulation of inflammation markers [30].

Autoantibodies have been detected in patients with behavioral disorders, such as autism [27]. Some studies demonstrated improvement of patients suffering from autism by treatment with intravenous immunoglobulin (IVIG) as shown by a recent pilot study [5]. Autoimmunity to the central nervous system (CNS) may play a pathogenic role in a subgroup of patients with autism. This study aimed to assess plasma anti-ganglioside M1 autoantibodies levels in autistic children and comparing them with normal healthy children.

In our study, the ratio of male to female autistic children who were recruited randomly from the autism clinic in Cairo University Hospitals was about 5 to 1, which is nearly correlated with the study of Ahmedand Abou El-Seoud [1] who found that the ratio of male to female was ≈ 4 to 1. Also, Wiśniowiecka-Kowalnik and Nowakowska [32] have mentioned in their study that autism is one of the most prevalent groups of neurodevelopmental disorder that affects around 1–2% of the population with an average male to female ratio of 4–5:1. However, the ratio found in the current study is not representative of the general population due to the small sample size.

In the current study, the mean plasma anti-ganglioside M1 autoantibodies level was significantly higher in 40 autistic patients (3.75 ± 0.62 ng/ml) than in 40 sex- and age-matched control subjects (1.96 ± 0.3 ng/ml) (*P* value *= 0.001*). This is in agreement with a study conducted by Yang et al. [33] who found that autistic children had higher positive levels of anti-ganglioside M1 antibodies (37.8%) than controls (21.67%) (*P* value = 0.04). Also in line with the results of our study, Mostafa and Al-Ayadhi [17] observed that autistic children had significantly higher serum levels of anti-ganglioside M1 antibodies than healthy controls *(P* value *< 0.001).*

Also, suggesting a possible immunological process in autism, Kern et al. [14] found that brain autoantibody levels in autistic children were higher than those in the control group. Lekman et. al. [15] carried out a study on CSF samples obtained from 20 children with autism and 25 controls. He found that anti-ganglioside M1 autoantibodies were significantly increased in patients with autism compared with age-matched controls which is also concordant with the current study.

Careaga et al. [4] stated that increased anti-phospholipid antibody levels in young children with autism and the association between antibody levels and impaired behaviors in the pediatric population as whole offer potential new targets for understanding the mechanisms involved in the pathogenicity of autism.

Mazur-Kolecka et al. [16] tested the presence of autoantibodies against human neuronal progenitor cells in sera from children with autism (*n* = 20) and age-matched controls (*n* = 18) by immunoblotting and immunocytochemistry. They found that sera from individuals with autism had a higher incidence of autoantibodies than in control sera. Mostafa and Al-Ayadhi[18] also found that autistic children had a significantly higher percentage of serum anti-neuronal antibodies (62.5%) than healthy controls (5%) (*P* value ≤ 0.001). In addition to the aforementioned studies, a study conducted by Wills et al. [31] proved that there were autoantibodies against certain neural antigens in autistic patients compared to a normal healthy control group. Also, a study presented by Mostafa and Kitchener [19] mentioned that children with autism had significantly higher percent seropositivity of anti-nuclear antibodies than healthy children (*P* value ≤ 0.01).

Moreover, a study conducted by [28], that included 30 normal and 68 autistic children using immunoblotting assay, found that autistic children but not normal children had antibodies to the caudate nucleus (49% positive sera), cerebral cortex (18% positive sera) and cerebellum (9% positive sera) [26] analyzed autoantibody repertoires to brain tissue extract in the plasma of 171 autistic children and 54 controls. They showed statistically significant higher levels in children with autism than in the control group. In addition, [29] found a significant increase in the incidence of (IgG isotype) to neuron-axon filament protein and glial fibrillary acidic protein in autistic subjects. In a cohort study conducted by [9], it was found that children with autism have a greater frequency of serum antibodies to brain endothelial cells and their nuclei than healthy children. The presence of these antibodies raises the possibility that autoimmunity plays a role in the pathogenesis of language and social developmental abnormalities in a subset of children with these disorders.

On other hand [3] carried out a study on 42 children (24 males, 18 females) with autism in comparison to 21 (13 males, 8 females) healthy-matched children aged between 2 and 12 years. There was no seropositivity of anti-neuronal antibodies in either of the groups including anti-ganglioside M1 autoantibodies. A possible cause for the discrepancy between the result of this study and our study is that autism is not considered as a typical autoimmune disease, self-reactive antibodies or autoantibodies against a wide variety of targets have been found in a subset of patients with autism. In addition, another study conducted by [22] suggested that there was no consistent evidence to indicate that the reactivity of plasma from children to neural profiles in the brain is related to an autism diagnosis. Also, in another study by [23], no difference was found in the rate of occurrence of autoantibodies to cerebellar golgi neurons between children with autism and healthy peers. This finding was not concordant with the current study and the discrepancy may be attributed to the different type of autoantibodies to different target brain tissue measured in both studies and also due to the difference in the technique used, where coronal sections of brain tissue from a rhesus macaque (*Macaca mulatta*) were used for immunohistochemical analysis in the latter study.

In the present work, children with severe autism had slightly higher levels of anti-ganglioside M1 antibodies (3.77 ± 0.68 ng/ml) than those patients with mild to moderate autism (3.73 ± 0.57 ng/ml), but this difference was statistically insignificant (*P* value = 0.840). On the other hand, [17] have found that anti-ganglioside M1 antibodies levels had significant positive correlations with the degree of the severity of autism which may be due to the larger sample size in the latter study. Also, [14] found that brain autoantibody levels in autistic patients showed significant correlation with autism severity. The children in the latter study were all exposed to mercury that may have elicited the production of autoantibodies, unlike the present study; therefore, this may have led to different results.

Also, in the study conducted by [18], the frequency of the positivity of serum anti-neuronal antibodies was significantly higher in children with severe autism than children with mild to moderate autism. This finding was not concordant to the results of the present study, probably because the sample size in the latter study was larger (80 patients with autism compared to 80 healthy controls) which may have also contributed to the difference in results.

Further studies with larger sample size as well as multi-center studies are required to delineate the relation between GM1 and disease severity. Also more studies are required to assess and follow-up the effect of different autoimmune therapeutic strategies in treating patients with autism.

### Strengths and limitations

The strength in this study is that it evaluates the relation of anti-ganglioside M1 autoantibodies level and the severity of autism in a sample of Egyptian children, not merely its level without relating it to the clinical picture. Among the study limitations is the reduced clinical input which was largely due to global COVID-19 pandemic. In addition, the sample taken was a convenient sample which is another limitation. This study is a cross-sectional study, however a follow up study would more clearly show the relation between the level of anti-ganglioside M1 autoantibodies and autism clinical course over time.

## Conclusions

Plasma anti-ganglioside M1 levels is higher in autistic patients than in healthy controls which may implicate that some cases of autism may be autoimmune in nature. Plasma anti-ganglioside M1 antibodies levels may be related to the severity of the clinical picture in children with autism, as the children in this study who were suffering from severe autism showed higher anti-ganglioside M1 antibodies plasma levels than those suffering from mild to moderate autism, though the difference was statistically insignificant.

## Data Availability

The datasets used and or analyzed during the current study are available from the corresponding author on reasonable request.

## Abbreviations

CARS: Childhood Autism rating scale
CNS: Central nervous system
CSF: Cerebrospinal fluid
DSM IV-TR: Diagnostic and Statistical Manual of Mental Disorders, 4th Edition, text revised
EDTA: Ethylenediamine tetraacetic acid
ELISA: Enzyme–linked immunoassay
IVIG: Intravenous immunoglobulin
SSPS: Statistical Package for the Social Sciences

## References

[1] Ahmed S, Abou El-Seoud S (2019) Emotion intelligence enhancement and improvement for people with autism. J Adv Inf Technol 10(1):29–34

[2] American Psychiatric Association (2000). Diagnostic criteria from DSM-IV-TR.

[3] Bayram A, Kardas F, Demirci E, Gökahmetoğlu S, Özmen S, Canpolat M, Oztop D, Kumandaş S, Gümüş H, Per H (2016). Lack of serum antineuronal antibodies in children with autism. Bratisl Lek Listy.

[4] Careaga M, Hansen R, Hertz-Piccotto I, Van De Water J, Ashwood P (2013). Increased anti- phospholipid antibodies in autism spectrum disorders. J Neuroinflammation.

[5] Carpita B, Marazziti D, Palego L, Giannaccini G, Betti L, Dell'Osso L (2020). Microbiota, immune system and autism spectrum disorders: an integrative model towards novel treatment options. Curr Med Chem.

[6] Chan Y (2003). Biostatistics102: quantitative data – parametric and non-parametric tests. Singapore Med J.

[7] Chan Y (2003). Biostatistics 103: qualitative data –tests of independence. Singapore Med J.

[8] Chan Y (2003). Biostatistics 104: correlational analysis. Singapore Med J.

[9] Connolly A, Chez M, Pestronk A, Arnold S, Mehta S, Deuel R (1999). Serum autoantibodies to brain in Landau-Kleffner variant, autism, and other neurologic disorders. J Pediatr.

[10] Cutillo G, Saariaho A, Meri S (2020). Physiology of gangliosides and the role of antiganglioside antibodies in human diseases. Cell Mol Immunol.

[11] Elamin N, Al-Ayadhi (2014). Brain autoantibodies in autism spectrum disorder. Biomark Med.

[12] He P, Guo C, Wang Z, Chen G, Li N, Zheng X (2018). Socioeconomic status and childhood autism: A population-based study in China. Psychiatry Res.

[13] Hyman S, Levy S, Myers S (2020). Identification, evaluation, and management of children with autism spectrum disorder. Pediatrics.

[14] Kern J, Geier D, Mehta J, Homme K, Geier M (2020). Mercury as a hapten: A review of the role of toxicant-induced brain autoantibodies in autism and possible treatment considerations. J Trace Elem Med Biol.

[15] Lekman A, Skjeldal O, Sponheim E (1995). Gangliosides in children with autism. Acta Paediatr.

[16] Mazur-Kolecka B, Cohen I, Gonzalez M, Jenkins E, Kaczmarski W, Brown W, Flory M, Frackowiak J (2014). Autoantibodies against neuronal progenitors in sera from children with autism. Brain Dev.

[17] Mostafa G, Al-Ayadhi L (2011). Increased serum levels of anti-ganglioside M1 auto-antibodies in autistic children: relation to the disease severity. J Neuroinflammation.

[18] Mostafa G, Al-Ayadhi L (2012). The relationship between the increased frequency of serum antineuronal antibodies and the severity of autism in children. Eur J Pediatr Neurol.

[19] Mostafa G, Kitchener N (2009). Serum anti-nuclear antibodies as a marker of autoimmunity in Egyptian autistic children. Pediatr Neurol.

[20] Pape K, Tamouza R, Leboyer M, Zipp F (2019). Immunoneuropsychiatry novel perspectives on brain disorders. Nat Rev Neurol.

[21] Perry A, Condillac R, Freeman N, Geier J, Belair J (2005). Multi-site study of the Childhood Autism Rating Scale (CARS) in five clinical groups of young children. J Autism Dev Disord.

[22] Rossi C, Fuentes J, Van de Water J, Amaral G (2014). Brief report: antibodies reacting to brain tissue in Basque Spanish children with autism spectrum disorder and their mothers. J Autism Dev Disord.

[23] Rossi C, Van de Water J, Rogers S, Amaral D (2011). Detection of plasma autoantibodies to brain tissue in young children with and without autism spectrum disorders. Brain Behav Immun.

[24] Rossignol D, Genuis S, Frye R (2014). Environmental toxicants and autism spectrum disorders: a systematic review. Transl Psychiatry.

[25] Schopler E, Reichler R, Rochen Renner B (1988). The childhood autism rating scale.

[26] Silva S, Correia C, Fesel C, Barreto M, Coutinho M, Marques C, Miguel T, Ataide A, Bento C, Borges L, Oliveira G, Vicente A (2004). Autoantibody repertoires to brain tissue in autism nuclear families. J Neuroimmunol.

[27] Singer H, Morris C, Gause J, Gillin P, Crawford S, Zimmerman A (2008). Antibodies against fetal brain in sera of mothers with autistic children. J Neuroimmunol.

[28] Singh V, Rivas W (2004). Prevalence of serum antibodies to caudate nucleus in autistic children. Neurosci Lett.

[29] Singh V, Warren R, Averett R, Ghaziuddin M (1997). Circulating autoantibodies to neuronal and glial filament proteins in autism. Pediatr Neurol.

[30] Vargas D, Nascimbene C, Krishnan K, Zimmerman A, Pardo C (2005). Neuroglial activation and neuroinflammation in the brain of patients with autism. Ann Neurol.

[31] Wills S, Rossi C, Bennett J, Cerdeño V, Paul Ashwood P, Amaral D, Water J (2011). Further characterization of autoantibodies to GABAergic neurons in the central nervous system produced by a subset of children with autism. Mol Autism.

[32] Wiśniowiecka-Kowalnik B, Nowakowska B (2019). Genetics and epigenetics of autism spectrum disorder—current evidence in the field. J Appl Genet.

[33] Yang X, Liang S, Wang L, Han P, Jiang X, Wang J, Hao Y, Wu L (2018). Sialic acid and anti-ganglioside antibody levels in children with autism spectrum disorders. Brain Res.

[34] Zou T, Liu J, Zhang X, Tang H, Song Yand Kong X (2020). Autoantibody and autism spectrum disorder: a systematic review. Res Autism Spectr Disord.

